# The Association between Food Insecurity and Insomnia Symptoms among Young Adults in Puerto Rico and the Mediating Role of Psychological Distress Symptoms

**DOI:** 10.3390/ijerph21101296

**Published:** 2024-09-28

**Authors:** Natalia Vázquez-Colón, Andrea López-Cepero, Claudia Amaya, Katherine L. Tucker, Catarina I. Kiefe, Sharina D. Person, Milagros C. Rosal, Cynthia M. Pérez

**Affiliations:** 1 Department of Biostatistics and Epidemiology, Graduate School of Public Health, University of Puerto Rico, Medical Sciences Campus, San Juan, PR 00936, USAclaudia.amaya@upr.edu (C.A.); 2 Rollins School of Public Health, Emory University, Atlanta, GA 30322, USA; 3 Department of Biomedical and Nutritional Sciences, Zuckerberg College of Health Sciences, and Center for Population Health, University of Massachusetts Lowell, Lowell, MA 01854, USA; katherine_tucker@uml.edu (K.L.T.); 4 Department of Population and Quantitative Health Sciences, University of Massachusetts Chan Medical School, Worcester, MA 01655, USA; catarina.kiefe@umassmed.edu (C.I.K.); sharina.person@umassmed.edu (S.D.P.); milagros.rosal@umassmed.edu (M.C.R.)

**Keywords:** insomnia symptoms, food insecurity, psychological distress, Puerto Rico

## Abstract

Residents of Puerto Rico face a high burden of food insecurity (FI), which has been associated with insomnia symptoms (IS). However, this association remains understudied in Puerto Rican young adults, a vulnerable group experiencing an elevated prevalence of FI and poor sleep. We evaluated the association between FI and IS and the mediating role of psychological distress symptoms among young adults in Puerto Rico. Data are derived from the PR-OUTLOOK cohort (2020–2023) of adults aged 18–29 y. We assessed FI with the six-item USDA Household Food Security Scale and IS with the 5-item Women’s Health Initiative Insomnia Rating Scale. Psychological distress symptoms included depressive symptoms (CES-D-10), anxiety (STAI-10), and perceived stress (PSS-4). Poisson’s regression models estimated prevalence ratios (PRs) with 95% confidence intervals (CIs). The Karlson–Holm–Breen method estimated the mediation percentage of each psychological distress symptom on the association between FI and IS. Notably, 24.8% of participants experienced FI, and 30.4% reported elevated IS. FI was associated with IS (PR = 1.41, 95% CI = 1.24, 1.60), an association partially mediated by depressive (31.6%), perceived stress (17.6%), and anxiety symptoms (17.2%), accounting for 35.8% of the mediation percentage. Future research should confirm these findings using objective assessments of sleep and psychosocial stress.

## 1. Introduction

Food insecurity (FI) is defined as having restricted or unpredictable access to nutritious and safe foods or lacking reliable means to obtain acceptable foods through socially acceptable methods [1]. FI is a significant public health concern affecting 29.6% of the global population [2] and 12.8% of US households [3]. It disproportionately affects underrepresented groups in the US, with Hispanic households (20.8%) bearing a greater burden, compared to White, non-Hispanic households (9.8%) [3]. Puerto Rico is no exception, with 33.2% of the population aged 18 and older affected by FI [4]. Thus, with 40.7% of young adults in Puerto Rico living under the poverty line [5], there is a pressing need to understand FI and its potential association with other risk factors for chronic diseases in this vulnerable and understudied group, as it may affect their overall health and well-being.

FI is associated with adverse health outcomes [6,7,8], including insomnia symptoms (IS) [9,10,11]. Insomnia, characterized by difficulties in falling or staying asleep, waking up too early, or trouble returning to sleep after waking, affects overall sleep quality. IS impact about one-third of American adults aged 18 and older [11], disrupting their ability to achieve restful sleep. Despite this, few studies have examined the association between FI and IS in young adult populations [12,13,14]. Existing research supports associations between FI and IS [12] as well as poor sleep quality [13,14]. Young adults, who often face educational transitions and economic instability, are particularly vulnerable to both FI [10,12,13,14,15,16] and IS [12,17,18,19].

FI is a distressing life experience that could influence IS through various interconnected pathways, including psychological and nutritional mechanisms. The stress associated with FI may trigger psychological responses that disrupt sleep patterns [20], while nutritional deficiencies associated with FI may also contribute to poor mental health [21]. Psychological distress may mediate the association between FI and IS, as the emotional strain from FI increases stress levels, leading to poor mental health and sleep disturbances.

Most research on FI and IS has primarily focused on adults in general [10,22,23], highlighting the need to investigate these dynamics in young adults. Studies in the US have documented associations between FI and psychological distress [12,14,15,16] among young adults. Furthermore, psychological distress has been linked to insomnia among college [17,24] students. Yet, this association remains underexplored among young Puerto Rican adults—a high-risk population with elevated prevalence of FI (27%) [4], poor sleep quality (36.5%) [25], and symptoms of psychological distress (24% anxiety and 60% depression) [26]. Thus, the objectives of this study were two-fold: (1) examine the association between FI and IS in a sample of young adults aged 18–29 in Puerto Rico and (2) assess the mediating role of psychological symptoms in the association between FI and IS. We expected that FI would be positively associated with IS and that this association would be partially mediated by psychological symptoms.

## 2. Materials and Methods

### 2.1. Study Design and Participants

The current cross-sectional analysis used data from the Puerto Rico Young Adults’ Stress, Contextual, Behavioral & Cardiometabolic Risk (PR-OUTLOOK) study. Our sampling, eligibility, and recruitment approach have been described elsewhere [27]. Briefly, participants in the study were required to meet the following criteria: age between 18 and 29 years, living in Puerto Rico and self-identifying as Puerto Rican or with at least one parent born in Puerto Rico, no history of cognitive, physical, or mental health conditions, and not serving in the military. Participants were recruited between 2020 and 2023 using multiple strategies, including traditional (television and radio) and social media announcements, press releases, email and print advertisements, participants’ referrals, and other community outreach. After eligible individuals provided informed consent, they completed a baseline survey (online or by phone) followed by a clinic visit, during which research staff were available to clarify questions.

### 2.2. Measures

#### 2.2.1. Food Insecurity Status

FI was assessed using the six-item US Department of Agriculture Household Food Security Scale [28]. This questionnaire evaluates household FI by inquiring about food access and behavior during the previous 12 months. The FI score was calculated by summing responses to all six items, resulting in scores ranging from 0 to 6. We chose to define food-secure individuals as those with high or marginal food security (scores 0 and 1) and food-insecure individuals as those with low or very low food security (scores 2 to 6), as few young adults had very low food insecurity (5%). The scale has a sensitivity of 92% and a specificity of 99.4%, indicating its high accuracy in correctly identifying households with and without FI [28] and has also been validated in Spanish [29]. We assessed the scale’s reliability using Cronbach’s alpha, which indicated good reliability (Cronbach’s α = 0.83) in this sample of young adults. 

#### 2.2.2. Insomnia Symptoms

IS over the past month were assessed using the 5-item Women’s Health Initiative Insomnia Rating Scale (WHIIRS) [30]. This concise tool asks participants to rate the frequency of IS (ranging from “not in the past 4 weeks” to “5 or more times a week”), including trouble falling asleep, waking up during the night, waking up too early in the morning, trouble returning to sleep after waking too early, and the overall sleep quality (from “very sound/restful” to “very restless”). Response options range from 0 to 4, resulting in a possible total score of 20, with a score greater than 9 indicating clinically significant IS [30]. Previous psychometric evaluations show that the WHIIRS has good reliability, with a mean alpha coefficient of 0.78 and a test–retest reliability of 0.96 for same-day administration [30]. This scale has also been successfully used among Hispanic/Latino adults of all ages, showing good internal consistency (Cronbach’s α = 0.832) [31]. In the current study, the WHIIRS tool also demonstrated good internal consistency (Cronbach’s α = 0.76).

#### 2.2.3. Psychological Distress Symptoms

Psychological distress was assessed via symptoms of depression, anxiety, and perceived stress. Depression symptoms were measured using the 10-item short form of the Center for Epidemiologic Studies Depression Scale (CESD-10) [32]. Participants were asked to indicate on a scale from 0 (rarely or none of the time) to 3 (most or all of the time) the frequency of depression symptoms, including depressed mood, feelings of guilt, worthlessness, helplessness, psychomotor retardation, appetite changes, and restless sleep experienced over the prior week. A total score between 0 and 30 was obtained by summing the item scores, with higher scores indicating greater depressive symptoms. The score was dichotomized at a cutoff of 10 or more, suggestive of symptoms with potential clinical significance [32]. The CESD-10 scale has been recommended for use in Hispanics/Latinos, with acceptable internal consistencies across ethnic background groups (Cronbach α’s = 0.80–0.86 for the Spanish version), including Puerto Ricans [33]. Anxiety symptoms were assessed through the 10-item Spielberger State-Trait Anxiety Inventory (STAI-10). Participants reported the frequency of anxiety-related symptoms (e.g., restlessness and nervousness) on a scale from 0 (almost never) to 3 (almost always), with total scores ranging from 10 to 40 [34]. To identify those with elevated anxiety levels, the scores were dichotomized at the upper quartile in the absence of a standard cutoff point. The STAI-10 scale has demonstrated internal consistency in Hispanics/Latinos (Cronbach’s α = 0.81 for the Spanish version) [35]. Perceived stress was assessed using the Perceived Stress Scale-4 (PSS-4), a 4-item questionnaire that evaluates individual’s feeling of control in important aspects of their lives, their confidence in tackling personal problems, whether they feel overwhelmed by difficulties, and their general sense of composure over the past month [36]. Response options for these items ranged from 0 (never) to 4 (very often), and the overall score varied from 0 to 16. This score was dichotomized at the upper quartile in the absence of a standard cutoff point. The PSS-4 has been demonstrated to be a useful tool for assessing stress perception among students at a Hispanic-serving university in the US, showing adequate internal consistency (Cronbach’s α = 0.76) [37]. In our study, the internal consistency coefficients for CESD-10, STAI-10, and PSS-4 were 0.84, 0.86, and 0.75, respectively.

#### 2.2.4. Covariates

Several covariates were included to control potential confounding based on prior studies examining the association between FI and IS [10,11,12,13,14]. These covariates included age (18–24 y, 25–29 y), sex assigned at birth (male, female), marital status (not married, married), educational attainment (high school or less, some college/associate, college graduate or higher), past-year vaping (yes, no), and body mass index (BMI: <25.0, 25.0–29.9, and ≥30.0 kg/m^2^). Childhood material deprivation (yes, no) was defined by participants’ responses to whether their families faced difficulties affording necessities [38]. Subjective social status was measured using the MacArthur Scale (low, high), which asks participants to rank their perceived social standing relative to others in Puerto Rico on a 10-rung ladder [39]. Higher scores reflect higher subjective social status. This measure has been recommended for young adults, whose socioeconomic status is still in flux, making traditional socioeconomic measures less accurate [40,41].

### 2.3. Statistical Analyses

#### 2.3.1. Analytic Sample

Between September 2020 and November 2023, 2808 young adults were enrolled in the PR-OUTLOOK study. Participants were excluded if they had incomplete data on variables of interest. The exclusions were due to incomplete data on BMI (*n* = 419), subjective social standing (*n* = 11), education (*n* = 2), material deprivation (*n* = 1), and sex at birth (*n* = 1), resulting in an analytical sample of 2374 young adults.

#### 2.3.2. Analytic Approach

Descriptive statistics were obtained for the overall analytic sample and by FI status (insecure vs. secure). Differences in the distribution of participant characteristics by FI status were assessed using the chi-square test. Poisson’s regression models with robust error variance were fit to estimate the association between FI and IS, adjusting for age, sex assigned at birth, marital status, educational attainment, subjective social status, childhood material deprivation, past-year vaping, and BMI. Results are presented as prevalence ratios (PR) with 95% confidence intervals (95% CI). For the mediation analysis, we utilized the Karlson, Holm, and Breen method to decompose the total effect of FI on IS into direct and indirect components [42], adjusting for the same covariates. The models assessed the potential mediated effect of each psychological distress measure (i.e., symptoms of perceived stress, anxiety, and depression) and the mediated effect of all measures considered jointly. The direct effect represents the effect of FI on IS that is only due to FI. In contrast, the indirect effect is the effect of FI on IS that is explained by psychological distress symptoms. The total effect reflects the estimate of the effect of FI on IS, and the proportion mediated indicates the fraction of this effect mediated by psychological symptoms. In sensitivity analysis, the CESD-10 scale was examined as a 9-item scale, excluding the sleep item (restless sleep), to test the robustness of the findings. All analyses were conducted using Stata Statistical Software Release 18.0 (Stata Corp LLC, College Station, TX, USA). All *p*-values are two-sided, and significance is denoted by *p* < 0.05.

## 3. Results

Of the 2374 young adults included in this study, 24.8% were food insecure. Compared to the food-secure group, food-insecure individuals were more likely to be female, married or living with their partner, report lower subjective social standing, have obesity, and have experienced childhood material deprivation and current psychological distress symptoms (Table 1).

A significantly higher proportion of food-insecure individuals also reported experiencing IS than their food-secure counterparts (41.9% and 26.7%, respectively) (Table 2). In the unadjusted model, participants who reported FI were more likely to have IS compared to the food-secure group (PR = 1.57, 95% CI: 1.39–1.77, *p* < 0.001). After adjustment for covariates, FI remained significantly associated with IS (PR = 1.41, 95% CI: 1.24–1.60, *p* < 0.001).

The mediation analysis showed that all three psychological distress measures partially mediated the association between FI and IS (Table 3). Depression symptoms mediated the largest effect (31.6%), followed by perceived stress symptoms (17.6%) and anxiety symptoms (17.2%). All three psychological distress measures explained 35.8% of the observed association. After excluding the sleep item from the CESD-10 scale, the sensitivity analysis confirmed that depression symptoms mediated the largest effect (25.4%), followed by perceived stress (17.6%) and anxiety (17.2%) symptoms. These three psychological measures explained 31.8% of the FI-IS association (Table S1).

## 4. Discussion

In the present study of 2374 Puerto Rican young adults, FI was significantly associated with IS, with symptoms of psychological distress partially mediating this association and depression symptoms mediating the largest effect. These findings highlight the effect that FI and psychological distress may have on the sleep health of young adults, emphasizing the need for early intervention efforts to prevent psychosocial distress and IS.

Overall, our findings that FI was associated with IS align with the current, yet scant, literature on young adults, largely focused on college students [12,13,14]. For instance, a study analyzing data from 14,786 US young adults aged 24–32 y (58% White and 25% African American) found that FI was associated with higher odds of IS, including trouble falling asleep and staying asleep [12]. Another study conducted among 17,686 college students (84% non-Hispanic White) across 22 universities in 12 US states found that food-insecure students had higher odds of poor sleep quality [13]. A more recent study, with a convenience sample of 134 college students (48.5% Hispanic/Latino), also found that FI was associated with poor self-reported sleep health [14]. Thus, our study adds to the limited body of literature examining FI and IS among young adults, focusing on those of Hispanic background.

Our mediation analysis showed that psychological distress symptoms partially mediated the association between FI and IS, with depression symptoms as the primary mediator. These findings align with the study by Jacob et al. [10], which examined adults (mean age = 43.8 y) living in six low- and middle-income countries. That study found that anxiety symptoms, perceived stress, and depression diagnosed according to the DSM-IV criteria) mediated a substantial proportion (43.3%) of the association. Two additional studies assessed the role of psychological distress in the cross-sectional association between FI and other sleep outcomes. For instance, Troxel et al. [22] found that psychological distress attenuated the association between FI and subjective sleep quality among African American adults (mean age = 56 y) from two socioeconomically disadvantaged neighborhoods; however, sleep quality was determined based on responses to one question asking participants to rate how well they slept last night. Additionally, a study among 121 Latino individuals with type 2 diabetes (mean age = 61 y) found that depressive symptoms, anxiety symptoms, and diabetes distress each mediated the association between FI and worse sleep quality [23]. Future research should assess the mediating role of psychological distress and other potential pathways through which FI is associated with IS in diverse young adult populations. This is crucial for developing targeted interventions and informing public health policies to alleviate FI and reduce psychological distress.

Our findings highlight elevated proportions of FI, perceived stress, and symptoms of depression, anxiety, and insomnia. The proportion of participants who reported FI is consistent with the island-wide prevalence reported among Puerto Rican adults aged 18–24 (27%) [4]. This is lower than the prevalence estimates observed among college students (30.8%–67.6%) [13,14,15,16] but higher than among young adults from the National Longitudinal Study of Adolescent to Adult Health (11%) [12]. The variation in prevalence found across the different surveys might partially be attributed to the differing survey tools used to assess FI (USDA 10-item [13] vs. 6-item [14,15] vs. 2-item [16] vs. single item [12]) and differences in the sample racial/ethnic composition of young adults. Several studies have examined sleep disturbances among young adults, particularly students, but fewer have focused on IS [12,17]. Nearly a third of our sample reported IS, which is comparable to findings by Nagata et al. [12], where 28.5% of young adults aged 24–32 had trouble falling asleep, and 32.0% had trouble staying asleep. Similarly, Mbous et al. [17] found that 26.4% of students at two large Midwestern universities experienced insomnia. Thus, further research is needed to address the burden of IS among young adults, especially considering the limited focus on this age group and its impact on overall health and long-term well-being. Additionally, the high frequency of psychological distress symptoms in this sample of young adults in Puerto Rico is consistent with previous studies among medical and nursing students on the island [43] and other investigations among Latin American, US Hispanic/Latino, and Spanish college students [44]. The high level of psychological distress in our sample may be explained by the numerous stress exposures experienced by Puerto Ricans in the past decade, including several natural disasters (e.g., Hurricanes Irma and María in 2017 followed by a 6.2-magnitude earthquake and a prolonged aftershock sequence in 2020), the COVID-19 pandemic, high levels of poverty relative to other US states and jurisdictions, the island’s financial crisis, and a strained healthcare system with limited medical providers and access to care [45]. Given the challenges young adults face in Puerto Rico, further investigation into their impact is essential. Public health efforts are needed at the individual, community, and policy levels to address the prevailing social, behavioral, and psychological determinants of health among Puerto Rican young adults.

Our findings should be interpreted within the context of the study’s limitations. The cross-sectional design limits our ability to infer causality or determine the directionality of the associations. IS may contribute to psychological distress, and vice versa, making it challenging to disentangle these associations. Reverse causation is also plausible, as sleep problems could lead to lower job performance and income, potentially resulting in FI. Additionally, both FI and IS were self-reported, which could be subject to recall and social desirability biases. FI was measured as a dichotomous variable (food secure vs. insecure), rather than as a polytomous variable capturing different levels of FI. The sample also had a slight overrepresentation of women, married individuals, and adults with lower education, which may limit the generalizability of our findings. However, Puerto Rican young adults, who face significant health disparities [45], are an underrepresented group in which FI-sleep associations are not well studied. Although we controlled the effect of multiple confounders, the possibility of residual confounding cannot be excluded. Notwithstanding these limitations, the large sample size of this cohort, comprised of an ethnically homogeneous group, expands our understanding of the association between FI and IS in this vulnerable population. We also used validated and widely recognized scales to assess FI and psychological distress symptoms, adding robustness to our findings. While the WHIIRS tool used to assess IS was originally developed and validated for postmenopausal women from diverse racial/ethnic backgrounds, it has been successfully used among Latino adults of all ages showing good internal consistency (Cronbach’s α = 0.832 for adults of diverse Hispanic background; Cronbach’s α = 0.78 for adults of Puerto Rican background) [30]. As shown in the WHIIRS validation study [30], in our sample there were significant correlations between the WHIIRS scores and psychological distress scores and in the predicted direction (e.g., depressive symptoms, r = 0.38; anxiety symptoms, r = 0.35; and perceived stress, r = 0.32; *p* < 0.0001 for all).

## 5. Conclusions

The current study supports a cross-sectional association between FI and IS among a large sample of young adults in Puerto Rico, with psychological distress symptoms acting as partial mediators. While these findings indicate an association, it is important to acknowledge that causality cannot be inferred from a cross-sectional study. Addressing FI, mental health, and sleep health remains a public health priority, as identified by the Healthy People 2030 initiative [46]. Interventions that target upstream determinants of physical and mental health are critical to improving overall well-being. Future research should examine the temporal association between FI and sleep health using longitudinal designs and objective assessments of sleep and psychosocial stress. Our data support the need for effective upstream interventions (i.e., policies supportive of food access, public awareness campaigns, and strategies for reallocation of resources) to improve access to affordable, nutritious food, reduce psychological distress, and improve sleep health among young adults.

## Figures and Tables

**Table 1:** Sociodemographic and behavioral characteristics of young adults in Puerto Rico by food security status: PR-OUTLOOK, 2020–2023 (*n* = 2374).

Characteristic	Overall	Food Insecure ^1^	Food Secure	*p* Value
	(*n* = 2374)	(*n* = 589, 24.8%)	(*n* = 1785, 75.2%)	
	*n* (%)	*n* (%)	*n* (%)	
Age, y				
18–24	1718 (72.4)	408 (69.3)	1310 (73.4)	0.053
25–29	656 (27.6)	181 (30.7)	475 (26.6)	
Sex at birth				
Male	925 (39.0)	198 (33.6)	727 (40.7)	0.002
Female	1449 (61.0)	391 (66.4)	1058 (59.3)	
Marital status				
Not married	2077 (87.5)	489 (83.0)	1588 (89.0)	<0.001
Married	297 (12.5)	100 (17.0)	197 (11.0)	
Educational attainment				
High school or less	823 (34.7)	198 (33.6)	625 (35.0)	
Some college/Associate	546 (23.0)	174 (29.5)	372 (20.8)	<0.001
College graduate or higher	1005 (42.3)	217 (36.8)	788 (44.2)	
Subjective social standing				
Low (≤5)	1425 (60.0)	443 (75.2)	982 (55.0)	<0.001
High (>5)	949 (40.0)	146 (24.8)	803 (45.0)	
Childhood material deprivation				
Yes	772 (32.5)	295 (50.1)	477 (26.8)	<0.001
No	1602 (67.5)	294 (49.9)	1308 (73.3)	
Past-year vaping				
Yes	630 (26.5)	171 (29.1)	459 (25.7)	0.11
No	1744 (73.5)	418 (70.9)	1326 (74.3)	
BMI (kg/m^2^)				
<25.0	1158 (48.8)	258 (43.8)	900 (50.4)	
25.0–29.9	562 (23.7)	134 (22.8)	428 (24.0)	0.001
≥30.0	654 (27.6)	197 (33.5)	457 (25.6)	
Depressive symptoms ^2^				
≥10	1405 (59.2)	430 (73.0)	975 (54.6)	<0.001
<10	969 (40.8)	159 (27.0)	810 (45.4)	
Anxiety symptoms ^3^				
≥27	650 (27.4)	227 (38.5)	423 (23.7)	<0.001
<27	1724 (72.6)	362 (61.5)	1362 (76.3)	
Perceived stress symptoms ^4^				
≥6	1551 (65.3)	466 (79.1)	1085 (60.8)	<0.001
<6	823 (34.7)	123 (20.9)	700 (39.2)	

^1^ Assessed with the six-item USDA Household Food Security Scale; ^2^ CESD-10; ^3^ 10-item State-Trait Anxiety Inventory; ^4^ Perceived Stress Scale-4.

**Table 2:** Prevalence ratio and 95% confidence interval of insomnia-related symptoms, by food insecurity among young adults in Puerto Rico, PR-OUTLOOK, 2020–2023 (*n* = 2374).

Food Insecurity	Insomnia Symptoms (%)	Unadjusted Model			Adjusted Model *		
		PR	95% CI	*p*-Value	PR	95% CI	*p*-Value
No	26.7	1.00	-	-	1.00	-	-
Yes	41.9	1.57	1.39–1.77	<0.001	1.41	1.24–1.60	<0.001

* The regression model was adjusted for age, sex assigned at birth, marital status, education, subjective social status, childhood material deprivation, vaping, and BMI; Abbreviations: PR = Prevalence Ratio; CI = Confidence Interval.

**Table 3:** Mediation analysis of the effect of perceived stress, anxiety, and depression symptoms in the association between food insecurity and insomnia symptoms, PR-OUTLOOK, 2020–2023 (*n* = 2374).

Mediator	Effect	PR (95% CI) *	*p*-Value	Mediation Percentage
Perceived stress symptoms	Total	1.43 (1.26–1.63)	<0.001	17.6
	Direct	1.35 (1.19–1.53)	<0.001	
	Indirect	1.07 (1.04–1.10)	<0.001	
Anxiety symptoms	Total	1.42 (1.25–1.61)	<0.001	17.2
	Direct	1.34 (1.18–1.52)	<0.001	
	Indirect	1.06 (1.03–1.09)	<0.001	
Depression symptoms	Total	1.45 (1.28–1.64)	<0.001	31.6
	Direct	1.29 (1.14–1.46)	<0.001	
	Indirect	1.12 (1.08–1.17)	<0.001	
All	Total	1.44 (1.28–1.63)	<0.001	35.8
	Direct	1.27 (1.12–1.43)	<0.001	
	Indirect	1.14 (1.09–1.19)	<0.001	

* The models were adjusted for age, sex assigned at birth, marital status, education, subjective social status, childhood material deprivation, vaping, and BMI; Abbreviations: PR = Prevalence Ratio; CI = Confidence Interval.

## Data Availability

Data will be available to researchers who provide a methodologically sound proposal approved by the study steering committee to achieve the aims outlined in the approved proposal. Data can be obtained through a signed data access agreement. The agreement can be obtained by emailing the contact principal investigator at milagros.rosal@umassmed.edu.

## Supplementary Materials

The following supporting information can be downloaded at: https://www.mdpi.com/article/10.3390/ijerph21101296/s1, Table S1: Mediation analysis of the effect of perceived stress, anxiety, and depressive symptoms in the association of food insecurity and insomnia symptoms after excluding the sleep item from the CESD-10 scale, PR-OUTLOOK, 2020–2023 (n = 2374).
