# Supplementary material for: The Association between Food Insecurity and Insomnia Symptoms among Young Adults in Puerto Rico and the Mediating Role of Psychological Distress Symptoms
Source: Int J Environ Res Public Health. 2024 Sep 28;21(10):1296. doi: 10.3390/ijerph21101296 (PMC11507534; doi:10.3390/ijerph21101296)
Supplement: Supplementary file 1 [file ijerph-21-01296-s001.zip › ijerph-3140352-supplementary.pdf]

Supplementary Table

Table S1. Mediation analysis of the effect of perceived stress, anxiety, and depressive symptoms in the association of food insecurity and insomnia symptoms after excluding the sleep item from the CESD-10 scale, PR-OUTLOOK, 2020-2023 (n = 2374).

Mediator	Effect	PR (95% CI)	*p*-Value	Mediation Percentage
Perceived stress symptoms	Total	1.43 (1.26 – 1.63)	<0.001	17.6
	Direct	1.35 (1.19 – 1.53)	<0.001	
	Indirect	1.07 (1.04 - 1.10)	<0.001	
Anxiety symptoms	Total	1.42 (1.25 - 1.61)	<0.001	17.2
	Direct	1.34 (1.18 - 1.52)	<0.001	
	Indirect	1.06 (1.03 - 1.09)	<0.001	
Depression symptoms	Total	1.44 (1.27 - 1.63)	<0.001	25.4
	Direct	1.31 (1.16 - 1.49)	<0.001	
	Indirect	1.10 (1.06 - 1.14)	<0.001	
All	Total	1.44 (1.28 – 1.63)	<0.001	31.8
	Direct	1.28 (1.13-1.45)	<0.001	
	Indirect	1.12 (1.08- 1.17)	<0.001	
